# Differences in carotid arterial morphology and composition between individuals with and without obstructive coronary artery disease: A cardiovascular magnetic resonance study

**DOI:** 10.1186/1532-429X-10-31

**Published:** 2008-06-12

**Authors:** Hunter R Underhill, Chun Yuan, James G Terry, Haiying Chen, Mark A Espeland, Thomas S Hatsukami, Tobias Saam, Baocheng Chu, Wei Yu, Minako Oikawa, Norihide Takaya, Vasily L Yarnykh, Robert Kraft, J Jeffrey Carr, Joseph Maldjian, Rong Tang, John R Crouse

**Affiliations:** 1 Department of Radiology University of Washington, Seattle, WA, USA; 2 Department of Internal Medicine, Wake Forest University School of Medicine, Winston-Salem, NC, USA; 3 Biostatistical Sciences, Wake Forest University School of Medicine, Winston-Salem, NC, USA; 4 Department of Surgery, University of Washington, Seattle, WA, USA; 5 Department of Clinical Radiology, Ludwig-Maximilians-University, Munich, Germany; 6 Department of Radiology, Beijing Anzhen Hospital, Capital Medical University, Beijing, PR China; 7 Department of Radiology Wake Forest University School of Medicine, Winston-Salem, NC, USA; 8 Department of Neurology, Wake Forest University School of Medicine, Winston-Salem, NC, USA

## Abstract

**Objective:**

We sought to determine differences with cardiovascular magnetic resonance (CMR) in the morphology and composition of the carotid arteries between individuals with angiographically-defined obstructive coronary artery disease (CAD, ≥ 50% stenosis, cases) and those with angiographically normal coronaries (no lumen irregularities, controls).

**Methods and results:**

191 participants (50.3% female; 50.8% CAD cases) were imaged with a multi-sequence, carotid CMR protocol at 1.5T. For each segment of the carotid, lumen area, wall area, total vessel area (lumen area + wall area), mean wall thickness and the presence or absence of calcification and lipid-rich necrotic core were recorded bilaterally. In male CAD cases compared to male controls, the distal bulb had a significantly smaller lumen area (60.0 ± 3.1 vs. 79.7 ± 3.2 mm^2^, p < 0.001) and total vessel area (99.6 ± 4.0 vs. 119.8 ± 4.1 mm^2^; p < 0.001), and larger mean wall thickness (1.25 ± 0.03 vs. 1.11 ± 0.03 mm; p = 0.002). Similarly, the internal carotid had a smaller lumen area (37.5 ± 1.8 vs. 44.6 ± 1.8 mm^2^; p = 0.006) and smaller total vessel area (64.0 ± 2.3 vs. 70.9 ± 2.4 mm^2^; p = 0.04). These metrics were not significantly different between female groups in the distal bulb and internal carotid or for either gender in the common carotid. Male CAD cases had an increased prevalence of lipid-rich necrotic core (49.0% vs. 19.6%; p = 0.003), while calcification was more prevalent in both male (46.9% vs. 17.4%; p = 0.002) and female (33.3% vs. 14.6%; p = 0.031) CAD cases compared to controls.

**Conclusion:**

Males with obstructive CAD compared to male controls had carotid bulbs and internal carotid arteries with *smaller *total vessel and lumen areas, and an increased prevalence of lipid-rich necrotic core. Carotid calcification was related to CAD status in both males and females. Carotid CMR identifies distinct morphological and compositional differences in the carotid arteries between individuals with and without angiographically-defined obstructive CAD.

## Background

Cardiovascular Magnetic resonance (CMR) is a non-invasive imaging modality that has enabled the assessment of both the morphological [1] and compositional characteristics of the carotid arterial wall. Via histological validation, multi-sequence high-resolution carotid CMR has been proven to characterize fibrous tissue, lipid-rich necrotic core, calcification, and hemorrhage in the human atherosclerotic plaque *in vivo *[2-4]. Moreover, the high reproducibility [5-7] of carotid CMR has made possible the effective quantitative assessment of plaque evolution [8] and the identification of compositional features associated with accelerated progression [9].

Studies with carotid CMR, however, have been limited to the evaluation of subjects with established carotid disease [10,11]. Although these and other carotid studies [12] have proven invaluable in the assessment of local plaque phenomena, the relationship between CMR identified carotid disease and atherosclerotic disease in other arterial beds has not previously been reported. Associations between the carotid artery and other vascular beds have been described in the ultrasound literature [13]. In particular, multiple investigations have successfully employed B-mode ultrasound to identify differences in carotid intima-media thickness (IMT) between individuals with and without coronary artery disease (CAD) [14,15].

We designed the **C**arotid **A**therosclerosis (**M**RI) **P**rogression **S**tudy (CAMPS) to evaluate the presence and progression of carotid atherosclerosis measured by CMR over 2 years in patients with and without obstructive CAD. CAMPS is an observational, prospective investigation and participants were not selected based on carotid status. In the study described herein, we evaluated baseline data from this cohort and tested the hypothesis that individuals with and without obstructive CAD differ in their carotid arterial wall morphology and composition as identified by high spatial resolution carotid CMR. We further sought to determine if these differences were unique to particular segments of the carotid artery and to identify any gender specific findings.

## Methods

### Patient population

Male and female individuals older than 45 years who had undergone cardiac catheterization were recruited for participation in CAMPS. Inclusion criteria for study enrollment were angiographically obstructive CAD (≥ 50% stenosis in one or more coronary artery, case group) or angiographically normal coronary arteries (no evidence of stenosis or lumen irregularities, control group). Individuals with non-obstructive coronary disease (stenosis of <50%) were excluded from enrollment and carotid CMR to optimize the potential of observing differences between CAD case and control groups. Additional exclusion criteria were the presence of clinical instability, untreated thyroid disease, liver disease, alcohol abuse, creatinine ≥ 2.5, pregnancy, or history of cerebrovascular disease intervention. We did not exclude from enrollment patients using lipid lowering agents or those with a history of coronary revascularization. We enrolled an equal number of male CAD cases and controls and female CAD cases and controls according to a stratified random sampling strategy to achieve an intended gender ratio of 1:1 in a fashion similar to that previously described [14]. Of note, the control population had no history of myocardial infarction and, in general, underwent catheterization for evaluation of chest pain, arrhythmia, or valvular heart disease. All participants provided answers to a standardized health questionnaire and had their height and weight recorded. The study was approved by the institutional review board prior to study initiation and all participants gave their written, informed consent.

### CMR protocol

Enrolled participants were imaged on a Signa 1.5T MRI scanner (General Electric Healthcare, Milwaukee, USA) using bilateral phased array carotid surface coils (Pathway MRI Inc., Seattle, USA). A previously published standardized protocol [16] was used to acquire four different contrast-weighted images (T1-weighted [T1W], proton density [PD], T2-weighted [T2W] and time of flight [TOF]) without the use of exogenous contrast agents. All images were obtained with a field of view of 16 cm and matrix size 256 × 256 for an in-plane acquisition resolution of 0.62 × 0.62 mm. Axial images of the bilateral carotid arteries were acquired with a 2 mm slice thickness over a longitudinal coverage of 32 mm. The more proximal of the two bifurcations was chosen as a reference, and 5 images were acquired above this bifurcation (internal carotid) and 10 below (carotid bulb and common carotid). Breath holding and electrocardiogram gating were not used, and total acquisition time was 30 min. After acquisition, all images underwent zero-filled interpolation to an image size of 512 × 512 resulting in pixel dimensions of 0.31 × 0.31 mm.

### Image review

**Figure 1:**
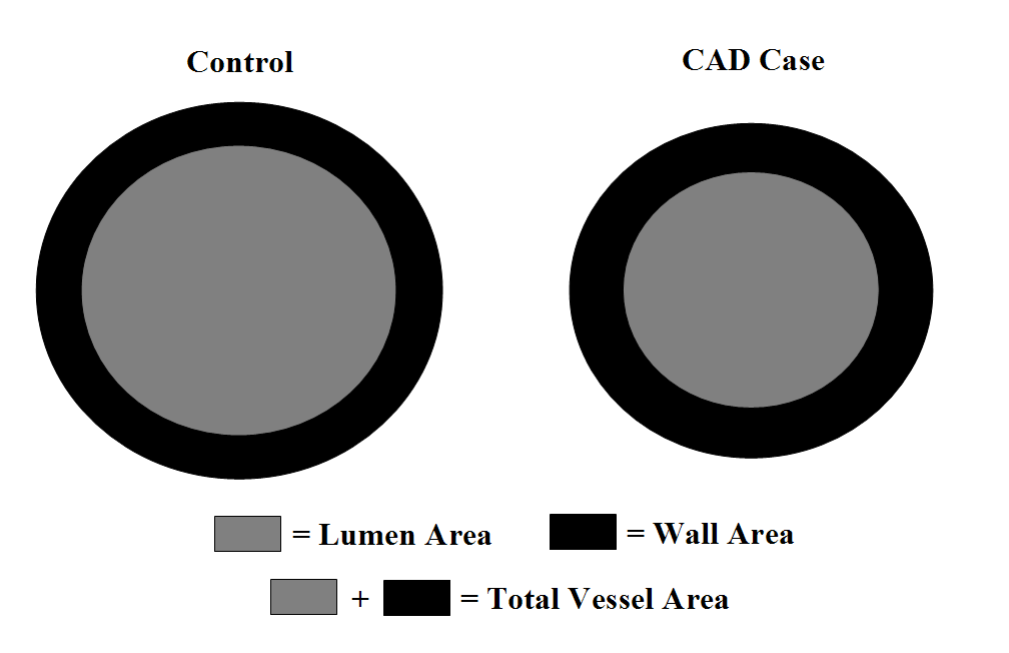
Illustration of the observed geometric differences between CAD cases and controls in the distal bulb and ICA. Dimensions of each shape were constructed in Matlab (The Mathworks, Natick, MA) to provide wall areas of identical size between shapes. Notice the increase in wall thickness in CAD cases associated with the smaller vessel size.

Two trained carotid CMR reviewers, blinded to CAD status and other participant characteristics, interpreted by consensus opinion both carotid arteries from each participant. Each artery was evaluated for image quality (4-point scale, 1 = poor, 4 = excellent). For arteries with image quality ≥ 2 image analysis software (CASCADE [17], Seattle, Washington, USA) was used to draw the lumen and outer wall boundaries at each axial location. Lumen area, wall area, total vessel area (lumen area + wall area; Figure 1), normalized wall index (NWI = wall area/total vessel area), and mean wall thickness were recorded. In addition, the presence or absence of lipid-rich necrotic core and calcification was also identified at each slice level using established multi-contrast imaging criteria [18].

### Data analysis

**Figure 2:**
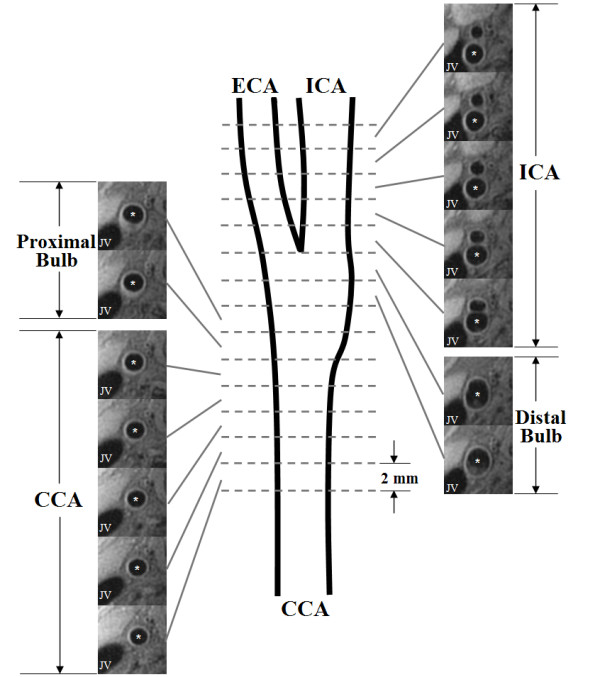
Schematic diagram of the coverage for each segment of the carotid artery. Each axial section had a 2 mm slice thickness with no inter-slice gap. Corresponding T1W images of a right carotid artery from each location are provided. CCA = common carotid artery; ECA = external carotid artery; ICA = internal carotid artery; JV = jugular vein; * = lumen of the specified segment.

For continuous measures (e.g. lumen area, wall area, total vessel area, etc) the mean of the left and right carotid artery for each arterial location determined by distance from the bifurcation was used during analysis. General linear mixed effects regression models were fitted by maximum likelihood to continuous measures to account for intra-patient correlations and the varying numbers of observed arterial slices among participants [19]. For each segment means and SEMs were calculated for both cases and controls. The results were compared for the whole population adjusting for gender, and separately by gender. Four vascular segments of the carotid artery were defined (Figure 2): 1) common: 10 – 20 mm proximal to the bifurcation; 2) proximal bulb: 6 – 10 mm proximal to the bifurcation; 3) distal bulb: 0 – 6 mm proximal to the bifurcation; and 4) internal: 0 – 10 mm distal to the bifurcation. For components, variables were summarized at the patient level such that a component was considered present if it was present on either artery. For each segment mean percent prevalence was reported for both cases and controls. Fisher's exact test was used to compare the results for the whole population, and separately by gender. Results were considered significant for *P *≤ 0.05.

## Results

From November 11, 2004 to February 23, 2006, 200 individuals were evaluated with carotid CMR. Secondary to motion artifact or poor image quality, 9 (4.5%) patients (3 CAD cases, 6 controls) were excluded. Table 1 summarizes the demographics of the 191 participants included in the analysis.

**Table 1:** Patient demographic information in mean ± SE or %.

**Characteristic**	**Female**		**Male**	
	
	**CAD Case **(N = 48)	**Control **(N = 48)	**CAD Case **(N = 49)	**Control **(N = 46)
**Age**, years	60.5 ± 1.4	60.8 ± 1.1	59.8 ± 1.1	57.8 ± 1.3
**Height**, cm	162.9 ± 1.0	160.2 ± 0.8	174.8 ± 0.9	174.3 ± 0.9
**Weight**, kg	75.8 ± 2.4	77.0 ± 2.3	89.4 ± 2.3	95.8 ± 2.4
**Race/Ethnicity**				
**Caucasian**, %	85.4	85.4	81.6	91.3
**African-American**, %	14.6	8.3	16.3	8.7
**Hispanic**, %	0	6.3	2.0	0
**Diabetes**, %	20.8	8.3	28.6	10.9
**Smoking**, %	54.2	35.4	62.5	54.3
**Hypertension**, %	72.9	56.3	77.6	52.2
**Statin Use**, %	77.1	25.0	85.7	19.6

### Arterial structure

For the group as a whole, the distal bulb and internal carotid artery (ICA) of CAD cases demonstrated the greatest differences compared to controls (Table 2). In CAD cases compared to controls, the distal bulb had a significantly smaller lumen area (54.3 ± 1.2 vs. 64.1 ± 1.3 mm^2^, p < 0.001) and total vessel area (90.1 ± 1.7 vs. 99.2 ± 1.7 mm^2^; p < 0.001) with a larger mean wall thickness (1.18 ± 0.02 vs. 1.09 ± 0.02 mm; p < 0.001) and NWI (0.409 ± 0.005 vs. 0.370 ± 0.005; p < 0.001), but comparable wall area (35.8 ± 0.6 vs. 35.0 ± 0.6 mm^2^; p = 0.4). Similarly, the ICA had a smaller lumen area (33.3 ± 1.2 vs. 38.4 ± 1.2 mm^2^; p = 0.003) and total vessel area (57.4 ± 1.6 vs. 62.4 ± 1.6 mm^2^; p = 0.03) with increased mean wall thickness (1.04 ± 0.02 vs. 0.97 ± 0.02; p = 0.002) and NWI (0.434 ± 0.005 vs. 0.400 ± 0.005; p < 0.005), and no difference in wall area (24.1 ± 0.6 vs. 24.0 ± 0.6 mm^2^; p = 0.9). Of note, the comparability of wall area between groups results from the combination of a smaller overall artery (lumen area and total vessel area) and greater wall thickness of the CAD cases compared to controls (Figure 1). In the common carotid artery, there were no significant between-group differences in any structural metric (Table 2).

**Table 2:** Comparison of arterial morphology fitted mean (SE) values after controlling for intra-subject correlations and gender for the whole sample population.

**Characteristic**	**CAD Cases **(N = 97)	**Controls **(N = 94)	***p-value***
**ICA**			
**LA**, mm^2^	**33.3 **± **1.2**	**38.4 ± 1.2**	**0.003**
**TVA**, mm^2^	**57.4 **± **1.6**	**62.4 ± 1.6**	**0.03**
**WA**, mm^2^	24.1 ± 0.6	24.0 ± 0.6	0.9
**MWT**, mm	**1.04 **± **0.02**	**0.97 ± 0.02**	**0.002**
**NWI**	**0.434 **± **0.005**	**0.400 ± 0.005**	**<0.001**
**Distal Bulb**			
**LA**, mm^2^	**54.3 **± **1.2**	**64.1 ± 1.3**	**<0.001**
**TVA**, mm^2^	**90.1 **± **1.7**	**99.2 ± 1.7**	**<0.001**
**WA**, mm^2^	35.8 ± 0.6	35.0 ± 0.6	0.4
**MWT**, mm	**1.18 **± **0.02**	**1.09 ± 0.02**	**<0.001**
**NWI**	**0.409 **± **0.005**	**0.370 ± 0.005**	**<0.001**
**Proximal Bulb**			
**LA**, mm^2^	37.6 ± 1.3	41.0 ± 1.3	0.06
**TVA**, mm^2^	64.6 ± 1.7	67.3 ± 1.7	0.3
**WA**, mm^2^	27.0 ± 0.6	26.4 ± 0.6	0.4
**MWT**, mm	**1.08 **± **0.02**	**1.03 ± 0.02**	**0.03**
**NWI**	**0.422 **± **0.005**	**0.404 ± 0.006**	**0.02**
**Common**			
**LA**, mm^2^	33.0 ± 1.2	33.4 ± 1.2	0.8
**TVA**, mm^2^	55.9 ± 1.6	55.5 ± 1.6	0.9
**WA**, mm^2^	22.9 ± 0.6	22.1 ± 0.6	0.3
**MWT**, mm	0.98 ± 0.02	0.95 ± 0.02	0.12
**NWI**	0.413 ± 0.005	0.404 ± 0.005	0.2

**ICA **= internal carotid artery; **LA **= lumen area; **TVA **= total vessel area; **WA **= wall area; **MWT **= mean wall thickness; **NWI **= normalized wall index

In gender specific analysis (Table 3), male CAD cases differed from controls in a fashion that was similar to differences found in the group as a whole. In male CAD cases compared to male controls, the distal bulb had a significantly smaller lumen area (60.0 ± 3.1 vs. 79.7 ± 3.2 mm^2^, p < 0.001) and total vessel area (99.6 ± 4.0 vs. 119.8 ± 4.1 mm^2^; p < 0.001) with an increased mean wall thickness (1.25 ± 0.03 vs. 1.11 ± 0.03; p = 0.002) and NWI (0.407 ± 0.009 vs. 0.349 ± 0.010; p < 0.001) and similar wall area (39.7 ± 1.2 vs. 40.0 ± 1.3 mm^2^; p = 0.4). Likewise, the ICA had a smaller lumen area (37.5 ± 1.8 vs. 44.6 ± 1.8 mm^2^; p = 0.006) and total vessel area (64.0 ± 2.3 vs. 70.9 ± 2.4 mm^2^; p = 0.04), larger NWI (0.442 ± 0.011 vs. 0.385 ± 0.011; p = 0.013), and no difference in wall area (26.5 ± 0.9 vs. 26.4 ± 0.9 mm^2^; p = 0.9). In contrast, there were no significant differences between female CAD cases and female controls in the distal bulb, and differences were only present for NWI in the ICA (0.440 ± 0.011 vs. 0.414 ± 0.010; p = 0.42). We detected no significant structural differences in the common carotid for either gender (Table 3), except for NWI between male groups (0.406 ± 0.006 vs. 0.387 ± 0.006; p = 0.03).

**Table 3:** Comparison of arterial morphology fitted mean (SE) values for each gender.

**Characteristic**	**Female**			**Male**		
	
	**CAD Cases **(N = 48)	**Controls **(N = 48)	***p-value***	**CAD Cases **(N = 49)	**Controls **(N = 46)	***p-value***
**ICA**						
**LA**, mm^2^	30.2 ± 1.9	32.3 ± 1.8	0.4	**37.5 ± 1.8**	**44.6 ± 1.8**	**0.006**
**TVA**, mm^2^	52.4 ± 2.4	54.1 ± 2.3	0.6	**64.0 ± 2.3**	**70.9 ± 2.4**	**0.037**
**WA**, mm^2^	22.1 ± 0.9	21.8 ± 0.8	0.8	26.5 ± 0.9	26.4 ± 0.9	0.9
**MWT**, mm	1.01 ± 0.03	0.94 ± 0.03	0.12	1.07 ± 0.03	0.99 ± 0.03	0.07
**NWI**	**0.440 ± 0.011**	**0.414 ± 0.010**	**0.042**	**0.422 ± 0.011**	**0.385 ± 0.011**	**0.013**
**Distal Bulb**						
**LA**, mm^2^	48.9 ± 3.2	49.2 ± 3.1	0.9	**60.0 ± 3.1**	**79.7 ± 3.2**	**<0.001**
**TVA**, mm^2^	80.5 ± 4.1	79.5 ± 4.0	0.9	**99.6 ± 4.0**	**119.8 ± 4.1**	**<0.001**
**WA**, mm^2^	31.7 ± 1.3	30.2 ± 1.2	0.9	39.7 ± 1.2	40.0 ± 1.3	0.4
**MWT**, mm	1.11 ± 0.03	1.06 ± 0.03	0.2	**1.25 ± 0.03**	**1.11 ± 0.03**	**0.002**
**NWI**	0.408 ± 0.009	0.391 ± 0.009	0.2	**0.407 ± 0.009**	**0.349 ± 0.010**	**<0.001**
**Proximal Bulb**						
**LA**, mm^2^	33.7 ± 2.0	31.1 ± 1.9	0.3	**41.9 ± 1.9**	**50.2 ± 2.0**	**0.003**
**TVA**, mm^2^	58.4 ± 2.7	53.8 ± 2.7	0.2	**71.4 ± 2.7**	**80.1 ± 2.8**	**0.02**
**WA**, mm^2^	24.7 ± 1.0	22.7 ± 1.0	0.13	29.5 ± 1.0	29.9 ± 1.0	0.7
**MWT**, mm	1.04 ± 0.03	0.99 ± 0.03	0.2	1.11 ± 0.03	1.06 ± 0.03	0.2
**NWI**	0.426 ± 0.008	0.424 ± 0.008	0.8	**0.414 ± 0.007**	**0.385 ± 0.008**	**0.007**
**Common**						
**LA**, mm^2^	30.0 ± 1.2	28.8 ± 1.2	0.4	35.9 ± 1.3	38.5 ± 1.3	0.2
**TVA**, mm^2^	51.9 ± 1.7	48.9 ± 1.7	0.2	60.3 ± 1.8	62.3 ± 1.9	0.4
**WA**, mm^2^	21.8 ± 0.7	20.2 ± 0.6	0.06	24.3 ± 0.7	23.8 ± 0.7	0.6
**MWT**, mm	0.97 ± 0.02	0.93 ± 0.02	0.09	1.00 ± 0.02	0.96 ± 0.02	0.15
**NWI**	0.421 ± 0.006	0.419 ± 0.006	0.8	**0.406 ± 0.006**	**0.387 ± 0.006**	**0.03**

**ICA **= internal carotid artery; **LA **= lumen area; **TVA **= total vessel area; **WA **= wall area; **MWT **= mean wall thickness; **NWI **= normalized wall index

### Arterial composition

For the entire evaluable population, patients with obstructive CAD had a significantly higher prevalence of carotid arterial calcification (40.2% vs. 16.0%, p < 0.001) and lipid-rich necrotic core (38.1% vs. 18.1%, p = 0.002) compared to controls. The prevalence of each component for each arterial segment is presented in Table 4. Of note, compositional differences between CAD cases and controls were most pronounced in the distal bulb and ICA.

**Table 4:** Comparison of plaque component prevalence for the whole sample population.

**Characteristic**	**CAD Cases **(N = 97)	**Controls **(N = 94)	***p-value***
**ICA**			
**Calcification**, %	**23.2**	**6.4**	**0.002**
**LRNC**, %	16.8	9.6	0.20
**Distal Bulb**			
**Calcification**, %	**32.0**	**11.7**	**<0.001**
**LRNC**, %	**29.9**	**12.8**	**0.004**
**Proximal Bulb**			
**Calcification**, %	**9.3**	**1.1**	**0.011**
**LRNC**, %	12.4	5.4	0.13
**Common**			
**Calcification**, %	2.0	0.0	0.5
**LRNC**, %	3.1	0.0	0.2

**ICA **= internal carotid artery; **LRNC **= lipid-rich necrotic core

The prevalence of arterial calcification was greater among CAD patients in both males (46.9% vs. 17.4%, p = 0.002) and females (33.3% vs. 14.6%, p = 0.031). The prevalence of lipid-rich necrotic core was greater among male and female CAD patients compared to controls, but this difference was significant only in males (males 49.0% vs. 19.6%, p = 0.003; females 27.1% vs. 16.7%, p = 0.22). We did not find any differences in plaque components in the common carotid or proximal bulb between groups for either gender (Table 5).

**Table 5:** Comparison of plaque component prevalence for each gender.

**Characteristic**	**Female**			**Male**		
	
	**CAD Cases **(N = 48)	**Controls **(N = 48)	***p-value***	**CAD Cases **(N = 49)	**Controls **(N = 46)	***p-value***
**ICA**						
**Calcification**, %	21.3	8.3	0.09	**25.0**	**4.3**	**0.007**
**LRNC**, %	14.9	8.3	0.3	18.8	10.9	0.3
**Distal Bulb**						
**Calcification**, %	**22.9**	**8.3**	**0.049**	**40.8**	**15.2**	**0.007**
**LRNC**, %	12.5	14.6	0.8	**46.9**	**10.9**	**< 0.001**
**Proximal Bulb**						
**Calcification**, %	10.4	2.1	0.2	8.2	0.0	0.11
**LRNC**, %	8.3	2.1	0.4	16.3	8.9	0.4
**Common**						
**Calcification**, %	2.1	0.0	0.3	2.1	0.0	0.3
**LRNC**, %	2.1	0.0	1.0	4.2	0.0	0.5

**ICA **= internal carotid artery; **LRNC **= lipid-rich necrotic core

**Figure 3:**
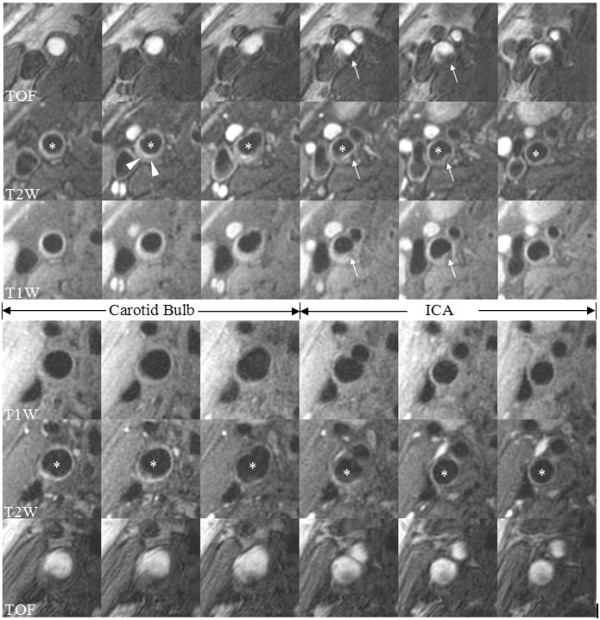
Comparison of multi-contrast MR images of the carotid bulb and ICA from a CAD case and control. The upper panel of images is from a 56 year old white male with obstructive CAD and the lower panel is from a 52 year old white male control. All images were acquired with the same imaging matrix and have been magnified identically. In the CAD case, notice the smaller total vessel area and lumen area and increased wall thickness. Also present in the distal bulb of the CAD case is a low T2W signal consistent with lipid-rich necrotic core (arrowheads). In the ICA, calcification (hypo-intensity on all image weightings, arrows) is present in the CAD case. The control has a normal appearing artery without any imaging evidence of a thickened wall or the presence of plaque components. The asterisk in the T2W sequence indicates the lumen of either the carotid bulb or ICA.

Figure 3 compares images from a male CAD patient to an age and gender matched control in order to illustrate the key morphological and compositional findings identified during this investigation.

## Discussion

This case-control study identified several new differences in carotid arterial structure and composition between patients with and without obstructive CAD. Males (but not females) with obstructive CAD had significantly smaller carotid arteries with thicker walls and an increased prevalence of lipid-rich necrotic core. These findings were limited to the carotid bulb and ICA. In contrast to these gender-specific differences, calcification was related to CAD status in both males and females.

The association between intima-media thickness of the carotid wall and CAD status has been well described in the ultrasound literature [13-15,20] and parallels our findings for CAD-related disparity in carotid wall thickness. In addition, however, we describe differences in area-based metrics (lumen area, total vessel area and NWI) between CAD cases and controls. The direction of these area-based differences was unexpected. From the study of coronary arteries, Glagov et al [21] proposed that during the expansion of early atherosclerotic lesions, preservation of luminal area occurred secondary to outward remodeling. The *smaller *total vessel area and lumen area that we detected in the CAD case group, while consistent with reported B-mode ultrasound measurements of ICA diameters from a similar cohort [22], appears inconsistent with the outward remodeling theory. This divergence is unlikely a result of the CAD cases having inward remodeling as a result of advanced carotid atherosclerotic disease since the wall area between groups was very similar. Without serial data to monitor the evolution of change, we can only speculate as to the etiology of the observed differences in arterial morphology. One possibility is that there may be inherent differences in arterial structure between the carotid arteries evaluated here and the coronary arteries that provided data for the remodeling hypothesis. Alternatively, the smaller carotid arteries of CAD cases may reflect impaired nitric oxide function manifested at a very early phase of atherosclerosis [23]. Lastly, individuals, particularly males, with smaller arteries at baseline (i.e. before they develop CAD or carotid disease) may be more prone to develop atherosclerotic disease. In this latter hypothesis, outward remodeling as predicted by Glagov may have resulted in the thickened arterial walls that were observed in the obstructive CAD cases.

A natural extension of the area-based metrics is NWI, which normalizes plaque burden to arterial size and is similar to the percent atheroma volume measurement proposed by Nissen et al for quantification of coronary atherosclerosis [24]. In previous CMR studies, NWI has been shown to be the most reproducible measure of plaque burden [5]. In this investigation, NWI provided the strongest discriminator of morphological differences between CAD cases and controls. Although there were differences in NWI at all segments between groups in males, the most robust differences in NWI and in the other metrics occurred in the distal bulb and ICA. Our findings suggest that early carotid disease may be best identified in the distal bulb and ICA. Furthermore, NWI may be the most sensitive CMR metric for detection of early atherosclerotic disease in any segment.

Except for NWI in the ICA, significant differences in arterial structure between groups were limited to males. Gender-based disparity in IMT is well recognized, but a gender-specific distinction in carotid structure between CAD cases and controls has not been previously reported. Crouse et al has previously documented a relationship between male gender and IMT progression rates in CAD cases [25]. These new observations indicate that carotid wall structure for this particular demographic may be more strongly associated with CAD in males than females.

Beyond structural measurements, an additional advantage of CMR is its ability to characterize arterial wall composition. In parallel with the differences between CAD cases and controls in arterial structure, presence of a lipid-rich necrotic core, particularly in the distal bifurcation, was associated with the presence of obstructive CAD in males. Integrated backscatter analysis in B-mode ultrasound has previously been used to demonstrate a difference in extent of carotid atheromatous tissue between patients with a history of myocardial infarction and low-risk controls [26]. These complimentary findings provide compelling evidence that the lipid-rich necrotic core is not only the basis of local carotid plaque instability [27], but that individuals with CAD may have an increased likelihood of developing plaques that have more pronounced lipid-rich necrotic cores.

Finally, calcification was a gender-independent marker and the most robust compositional indicator of obstructive CAD status. The association between calcification and cardiovascular disease has previously been demonstrated in the coronary arteries. Coronary artery calcification as measured by cardiac computed tomography has been closely correlated with severity of coronary artery disease at autopsy [28,29] and has been shown to predict cardiovascular events in asymptomatic individuals [30]. Our findings in the carotid artery are consistent with cardiac computed tomography data and indicate that the presence of calcification in either arterial bed may be indicative of CAD.

## Limitations

The strengths and limitations of using angiographically defined case-control groups have been previously described [31,32]. The control group may not accurately reflect an at-large population because of 1) the underlying reason for their referral to angiography, and 2) their proven absence of obstructive coronary disease. In addition, individuals with CAD generally receive intensive medical treatment by their primary care physician. Consequently, these results should not be extrapolated to the general population. Individuals with non-obstructive CAD (<50% stenosis) were not enrolled and did not receive a carotid CMR in this case-control study. In future investigations, this sub-group should be included since vulnerable coronary lesions have a high prevalence in non-obstructive CAD [33]. In so doing it will be possible to construct a more complete picture of the association between the structure and composition of the carotid artery and the angiographic severity of coronary disease. In addition, a prospective study including the full spectrum of CAD that aims to identify features or combinations of features in the carotid artery that predict future events should be conducted to establish the utility of this technology for risk stratification.

## Conclusion

We believe our study provides evidence for several conclusions. First, high spatial resolution CMR identifies distinct differences in both morphology and composition in the carotid arteries between patients with angiographically documented obstructive CAD and disease-free controls. Second, the distal bulb and ICA of males may be more susceptible to atherosclerotic disease than the common carotid or any segment of the carotid artery of females. Finally, the carotid artery may contain multiple indicators of CAD status that may prove useful during future investigations.

## Competing interests

The authors declare that they have no competing interests.

## Authors' contributions

HRU was in charge of the MR image review, collection and interpretation of data, and preparation of the manuscript. CY is a co-investigator of this study's grant and was in charge of CMR image protocol development and validation. He also assisted in data interpretation and manuscript revision. JGT assisted in development of the study design and interpretation of data. He also significantly revised the manuscript. HC, MAE and RT performed the primary analysis of the data and assisted in data interpretation. They also contributed to the drafting of the manuscript, particularly the section on statistical analysis. TSH assisted in data interpretation and was key to revising the critical content of the manuscript. TS, BC, NT, WY, and MO reviewed and peer-reviewed MR images. In addition, they assisted in data interpretation and manuscript revision. VY and RK assisted in image protocol development and study design. They also contributed primarily to the manuscript, particularly in the section describing the CMR protocol. JJC and JM were involved with study design and revision of the manuscript. JRC is the primary investigator of this study's grant. He was involved in all parts of this study and manuscript. All authors have read and approved submission of this manuscript. The material in the manuscript has not been published and is not being considered for publication elsewhere in whole or in part in any language.
